# Molecular surveillance of *pfcrt*, *pfmdr1* and *pfk13*-propeller mutations in *Plasmodium falciparum* isolates imported from Africa to China

**DOI:** 10.1186/s12936-021-03613-5

**Published:** 2021-02-06

**Authors:** Fang Huang, He Yan, Jing-Bo Xue, Yan-Wen Cui, Shui-Sen Zhou, Zhi-Gui Xia, Rabindra Abeyasinghe, Pascal Ringwald, Xiao-Nong Zhou

**Affiliations:** 1 National Institute of Parasitic Diseases, Chinese Center for Disease Control and Prevention, Chinese Center for Tropical Diseases Research, WHO Collaborating Center for Tropical Diseases, National Centre for International Research On Tropical Diseases, Ministry of Science and Technology, Key Laboratory of Parasite and Vector Biology, Ministry of Health, Shanghai, China; 2 World Health Organization Country Office in Philippines, Manila, Philippines; 3 World Health Organization, Geneva, Switzerland

**Keywords:** *Plasmodium falciparum*, Molecular markers, Drug resistance, Africa, China

## Abstract

**Background:**

The emergence and spread of multidrug resistance poses a significant risk to malaria control and eradication goals in the world. There has been no indigenous malaria cases reported in China since 2017, and China is approaching national malaria elimination. Therefore, anti-malarial drug resistance surveillance and tracking the emergence and spread of imported drug-resistant malaria cases will be necessary in a post-elimination phase in China.

**Methods:**

Dried blood spots were obtained from *Plasmodium falciparum*-infected cases returned from Africa to China between 2012 and 2015, prior to anti-malarial drug treatment. Whole DNA were extracted and known polymorphisms relating to drug resistance of *pfcrt*, *pfmdr1* gene, and the propeller domain of *pfk13* were evaluated by nested PCR and sequencing. The haplotypes and prevalence of these three genes were evaluated separately. Chi-squared test and Fisher's exact test were used to evaluate differences among the different sub-regions of Africa. A *P* value < 0.05 was used to evaluate differences with statistical significance. The maps were created using ArcGIS.

**Results:**

A total of 731 *P. falciparum* isolates were sequenced at the *pfcrt* locus. The wild type CVMNK was the most prevalent haplotype with prevalence of 62.8% and 29.8% of the isolates showed the triple mutant haplotype CV**IET**. A total of 434 *P. falciparum* isolates were successfully sequenced and *pfmdr1* allelic variants were observed in only codons 86, 184 and 1246. Twelve haplotypes were identified and the prevalence of the wild type *pfmdr1* NYD was 44.1%. The single mutant *pfmdr1* in codons 86 and 184 was predominant but the haplotype NY**Y** with single mutation in codon 1246 was not observed. The double mutant haplotype **YF**D was common in Africa. About 1,357 isolates were successfully sequenced of *pfk13*-propeller domain, the wild type was found in 1,308 samples (96.4%) whereby 49 samples (3.6%) had mutation in *pfk13*. Of 49 samples with *pfk13* mutations, 22 non-synonymous and 4 synonymous polymorphic sites were confirmed. The A578S was the most common mutation in *pfk13*-propeller domain and three mutations associated with artemisinin resistance (M476I, R539T, P553L) were identified in three isolates.

**Conclusion:**

This study provides evidence that could give insight into potential issues with anti-malarial drug resistance to inform national drug policy in China in order to treat imported cases.

## Background

Historically, malaria was one of the most serious infectious diseases in China. China has made great contributions towards global malaria control in the past 40 years. In 2010, China launched the National Malaria Elimination Programme (NMEP) 2010–2020 with the goal to interrupt local malaria transmission by 2020. Over the following five years, malaria cases decreased dramatically and there has been no indigenous malaria case reported since 2017 [1]. Now, China has achieved malaria elimination nationwide and is ready for World Health Organization (WHO) certification. However, with increasing globalization, larger numbers of people entering or returning from malaria-endemic areas present challenges to malaria elimination in China [2]. According to the national malaria report, there were more than 2500 imported cases annually, including over 100 patients with severe symptoms and approximately 10 deaths in 2017 and 2018 [3].

Over the past 50 years, *Plasmodium falciparum* has developed resistance to all anti-malarial drugs that have been used, including chloroquine (CQ), amodiaquine, sulfadoxine-pyrimethamine (SP), quinine, piperaquine, and mefloquine. Recently, the emergence and spread of multidrug resistance, including artemisinin and partner drug resistance of *P. falciparum* in Southeast Asia, poses a significant risk to malaria control and eradication goals in the world. The WHO had implemented a strategy to eliminate *P. falciparum* from the six countries in the Greater Mekong Sub-region (GMS) by 2025 to respond to the threat of an untreatable multidrug-resistant parasite [4]. Several mutations in the *P. falciparum* gene encoding a kelch protein on chromosome 13 (*pfk13*) are associated with artemisinin resistance [5] and have arisen multiple times and spread in the GMS. Over 200 non-synonymous *pfk13* mutations have been reported to date, of which nine validated variants (F446I, N458Y, M476I, Y493H, R539T, I543T, P553L, R561H, C580Y) and over 20 *pfk13* variants are considered as candidate mutations [6]. *Pfk13* mutations were detected predominantly in the GMS and were rare in Africa, but their profile was highly heterogeneous [5–7].

Mutations in *P. falciparum* CQ resistance transporter (*pfcrt*), located on the digestive vacuole membrane, were responsible for CQ resistance or treatment failure [8, 9]. Polymorphisms affecting amino acids at *pfcrt* residues 72–76 were observed in CQ-resistant field isolates, whereas *pfcrt* CVMNK haplotype was regarded as CQ-sensitive isolates [10, 11]. Polymorphisms in the *P. falciparum* multidrug-resistant 1 (*pfmdr1*) gene, encoding the plasmodial homologue of mammalian multidrug-resistant transporters, have previously been linked with anti-malarial drug resistance [12–15]*.* The mutations involving *pfmdr1* codons N86Y, Y184F, S1034C, N1042D, and D1246Y have been proven to be associated with mefloquine, lumefantrine, amodiaquine, CQ, and artemisinin, as well [16, 17].

Artemisinin-based combination therapy (ACT), which combines a fast-acting, rapidly eliminated artemisinin derivative with another slower-acting partner drug with a longer half-life, has been integral to the recent success of global malaria control. According to the current national malaria treatment policy in China, the first-line drugs to treat *P. falciparum* include three ACT (dyhidroartemisinin-piperaquine, artesunate-amadiquine, artesunate-piperaquine). Molecular surveillance of anti-malarial drug resistance markers is one of the tools to monitor and track the emergence and spread of drug resistance in imported malaria cases in China. This study collected the data of reported malaria cases from the national malaria case report system between 2012 and 2015, which were used to analyse malaria epidemiology in China. Dried blood spots were collected from *P. falciparum*-infected individuals returning from Africa in 2012–2015. The haplotypes of *pfcrt*, *pfmdr1* and *pfk13* genes were estimated by nested PCR and sequencing. The prevalence of different haplotypes of each gene was evaluated. The geographical distribution of the haplotypes of *pfcrt, pfmrd1* and *pfk13* genes in imported *P. falciparum* isolates from Africa were mapped.

## Methods

### Reported malaria cases

The data of all reported malaria cases, including indigenous and imported cases, were collected from the Chinese Infectious Disease Report System (CIDRS), a web-based reporting system between 2012 and 2015. Adhering to the ‘1-3-7’ strategy of the NMEP, a patient must be confirmed by microscope, rapid diagnostic test (RDT), or clinical test before the case was reported into the e-data system, and demographic data was recorded, including travel history, and/or imported source countries. According to the diagnostic criteria for malaria (WS 259-2015) in China, the clinically diagnosed cases were defined as patients with malaria-like symptoms and travel history to malaria-endemic areas but no parasites detected in blood examination. The epidemiology of imported malaria cases was analysed, and the main source countries were identified.

### Sample collection and DNA extraction

Dried blood spots on filter paper (Whatman™ 903, GE Healthcare, USA) were obtained from *P. falciparum*-infected cases who returned from Africa to China in 2012–2015 prior to anti-malarial drug treatment. Whole DNA was extracted from dry blood spots using a QIAamp DNA mini kit (Valencia, CA, USA) as described by the manufacturer. Microscopic examination of Giemsa-stained thick smears or RDT (Malaria HRP2/pLDH (P.f/Pan), Wondfo, Guangzhou, China) was used for malaria diagnosis within 24 h before the case was reported. Nested polymerase chain reaction (PCR), amplifying the small-subunit rRNA gene of *Plasmodium* spp. [18] was used to confirm the positive samples and the species before anti-malarial drug resistance markers were sequenced. Only samples with mono-infection of *P. falciparum* were sequenced in this study and samples with multiple infections were excluded.

### Nested PCR

The known polymorphisms relating to drug resistance at codons 72, 74, 75, 76 of the *pfcrt* gene and codons 86, 130, 184, 1034, 1042, 1109, 1246 of the *pfmdr1* gene, and also mutations on the propeller domain of the *pfk13* gene, were evaluated by nested PCR [5, 13, 19–21]. The primers for nested PCR, cycling conditions and sizes of PCR products are shown in Additional file 1. PCR products were purified using filter plates (Edge Biosystems, Gaithersburg, MD, USA) and directly sequenced and analysed on an ABI 3730XL automatic sequencer. The amplification products were analysed by 1.5% agarose gel electrophoresis before sequencing. Bi-directional sequencing was used and all the products were sequenced twice using independently amplified PCR products. The target amplified fragments covering polymorphic sites were as follows: amino acid position 51–83 for *pfcrt*, amino acid position 69–228 and 1030–1282 for *pfmdr1*, and amino acid position 433–702 for *pfk13*-propeller.

### Data analysis

The output sequence data were assembled, edited and aligned using Sequencher (version 5.1) software. All mutations were assessed by comparing each sequence to the 3D7 reference strain PF3D7_0709000 (*pfcrt*), PF3D7_0523000 (*pfmdr1*) and PF3DF_1343700 (*pfk13*) from PlasmoDB (http://www.plasmodb.org). The mixed alleles were determined according to the emergence of two chromatogram peaks at one nucleotide sited through the Mutation Surveyor (SoftGenetics LLC., version 5.1, State College, PA, USA). The prevalence of each haplotype was estimated by the number of the isolates carrying the specific haplotype and total samples with successful sequencing. R software (Version 4.0.2) and SAS software (SAS Institute Inc, Version 9.2, Cary, NC, USA) were used for data processing and statistical analysis. The Chi-squared test was used to evaluate differences among the different sub-regions but Fisher's exact test would be used if 25% of the cells had expected counts less than 5. A *P* value < 0.05 was used to evaluate differences with statistical significance. The maps were created by using ArcGIS 10.1 (Environmental Systems Research Institute, Inc, Redlands, CA, USA).

## Results

### Malaria epidemiology in China

**Fig. 1:**
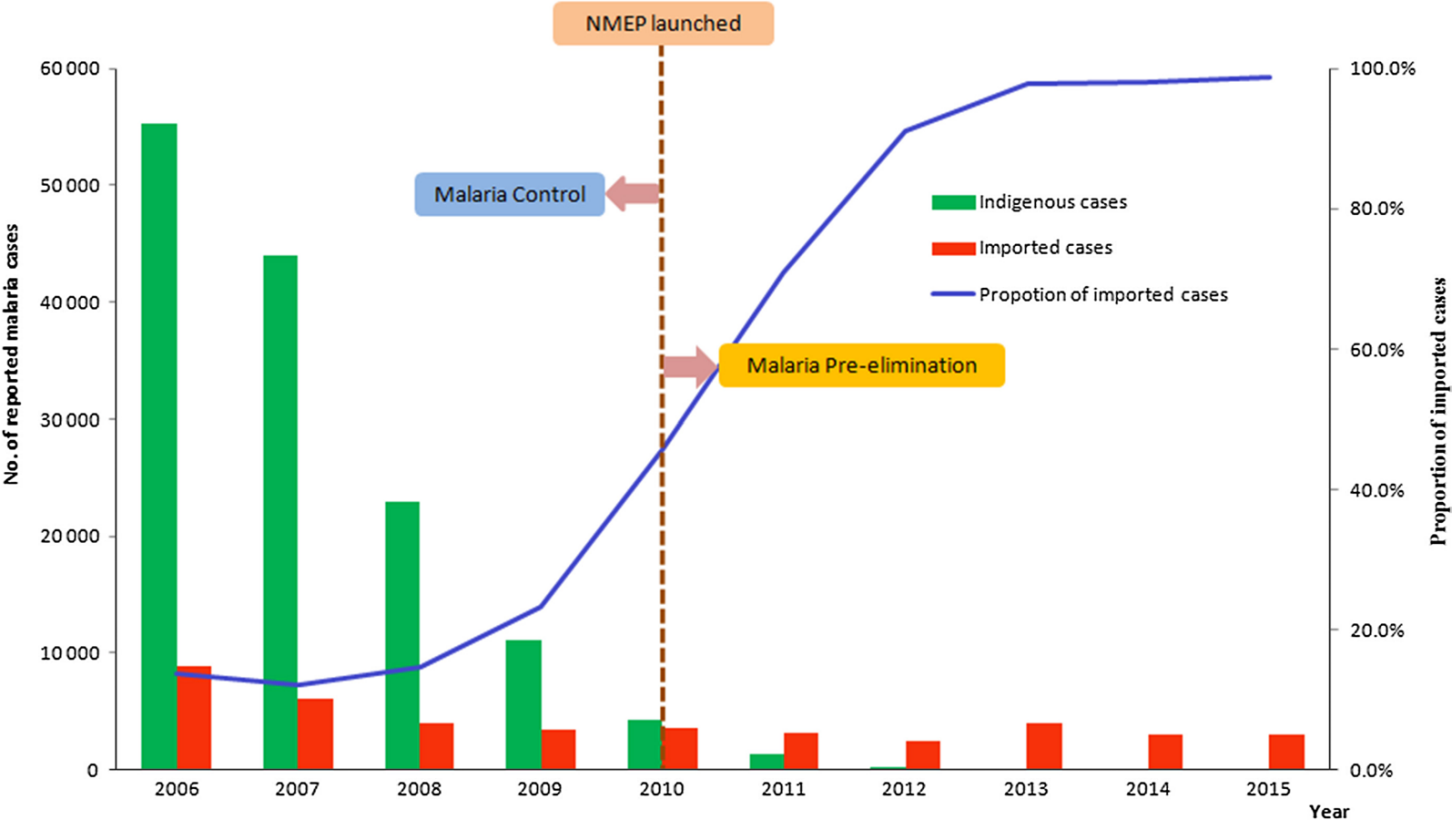
Indigenous and imported malaria cases reported in China from 2006–2015

The reported malaria cases decreased to only thousands of cases in 2012–2015 compared with hundreds of thousands cases before 2010, when the NMEP had not been launched. A total of 42 counties in the entire country reported indigenous cases in 2012 which decreased to nine counties in 2015 (Additional file 2). The proportion of imported cases has remained at more than 90% since 2012, and 244 indigenous cases were reported in 2012 (Fig. 1). Since 2013, the number of indigenous cases has dropped below 100 and most cases were reported from Yunnan and Tibet province/autonomous region in southern China. In 2017, no indigenous cases were reported in the country for the first time. Nevertheless, the proportion of imported *P. falciparum* cases increased from 2012 (n = 1403, 57.3%) to 2015 (n = 1895, 61.6%).

Imported cases originated from four continents and more than 70% were from central and western Africa. The main source countries of imported malaria cases in China are shown in Table 1. Ghana, Angola, Equatorial Guinea, and Nigeria have been the major source countries for malaria imported into China.

**Table 1:** Countries that are sources of imported malaria cases in China, 2012–2015

	Source country	Number and percentage of the imported malaria cases, n (%)				Total
		2012	2013	2014	2015	
1	Myanmar*	766 (31.0)	605 (15.0)	495 (16.4)	477 (15.5)	2343
2	Ghana	235 (9.5	1349 (33.4)	188 (6.2)	172 (5.6)	1944
3	Angola	151 (6.1)	437 (10.8)	272 (9.0)	416 (13.5)	1276
4	Equatorial Guinea	247 (10.0)	300 (7.4)	287 (9.5)	272 (8.8)	1106
5	Nigeria	207 (8.4)	225 (5.6)	341 (11.3)	283 (9.2)	1056
6	Cameroon	17 (0.7)	101 (2.5)	175 (5.8)	248 (8.1)	541
7	Democratic Republic of the Congo	47 (1.9)	64 (1.6)	118 (3.9)	175 (5.7)	404
8	Ethiopia	38 (1.5)	62 (1.5)	118 (3.9)	150 (4.9)	368
9	Guinea	58 (2.3)	75 (1.9)	64 (2.1	98 (3.2)	295
10	Indonesia^a^	36 (1.6)	71 (1.8)	142 (4.7	35 (1.1)	284
11	Republic of Congo	33 (1.3)	53 (1.3)	83 (2.7	101 (3.3)	270
12	Liberia	44 (1.8)	86 (2.1)	88 (2.9	34 (1.1)	252
	Other countries	595 (24.1)	614 (15.2)	650 (21.5)	616 (20.0)	2475
	Total	2474	4042	3021	3077	12,614

^a^Myanmar and Indonesia are two countries in Southeast Asia and the other 10 are countries in Africa

### Polymorphisms of *pfcrt*

A total of 731 *P. falciparum* isolates collected from imported cases form Africa were successfully sequenced at the *pfcrt* locus. Five haplotypes of *pfcrt* were identified including the wild type CVMNK, mutant haplotypes CVMN**T** and CV**IET,** mixed mutant haplotypes CVMN**K/T** and CV**M/I N/E K/T**. The wild type CVMNK was the most prevalent haplotype (62.8%, 459/731). The highest prevalence of CVMNK was eastern Africa (75.4%, 43/57) followed by northern Africa (73.3%, 11/15) and central Africa (62.4%, 251/402) (Fig. 1). There was no significant difference among the sub-regions of Africa (*P* = 0.2216). The single mutant type K76T was detected in four isolates and the mixed mutant type of K/T76 were only detected once from all the isolates. However, 29.4% (215/731) of the isolates carried the triple mutations CV**IET**. Two mixed mutant haplotypes were confirmed including CVMN**K/T** and CV**M/I N/E K/T** with the prevalence of 0.1% (1/731) and 7.1% (52/731), respectively. The distribution of polymorphisms and prevalence of different haplotypes of *pfcrt* are shown in Table 2.

**Table 2:** Geographic distribution of *pfcrt* and *pfmdr1* haplotypes in *Plasmodium falciparum* isolates returned from Africa

Gene	Haplotypes	Number and prevalence of each haplotype in different region of Africa (n/%)						
		East Africa	West Africa	Central Africa	South Africa	North Africa	Total	*P* value
*Pfcrt*	CVMNK (wild type)	43 (75.4)	101 (60.8)	251 (62.4)	53 (58.2)	11 (73.3)	459 (62.8)	0.2216
	CVMN**T**	3 (5.3)	0	1 (0.2)	0	0	4 (0.55)	0.01^b^
	CV**IET**	7 (12.3)	53 (31.9)	122 (30.3)	29 (31.9)	4 (26.7)	215 (29.4)	0.0598
	CVMN K/**T**	1 (1.8)	0	0	0	0	1 (0.1)	0.0985^b^
	CV M/**I** N/**E** K/**T**	3 (5.3)	12 (7.2)	28(7.0)	9 (9.9)	0	52 (7.1)	0.6406
Sub total		57	166	402	91	15	731	
*Pfmdr1*	NYD (wild type)	4 (20.0)	23 (32.4)	119 (46.3)	45 (52.3)	NA	191 (44.0)	0.0092
	**Y**YD^a^	0	2 (2.8)	16 (6.2)	5 (5.8)	NA	23 (5.3)	0.6466^b^
	N**Y**D	6 (30.0)	16 (22.5)	69 (26.8)	16 (18.6)	NA	107 (24.7)	0.417
	NY**Y**	0	0	0	0	NA	0	NA
	**YF**D	5 (25.0)	12 (16.9)	33 (12.8)	5 (5.8)	NA	55 (12.7)	0.0561
	**Y**Y**Y**	0	1 (1.4)	0	0	NA	1 (0.2)	NA
	N**FY**	0	0	1 (0.4)	0	NA	1 (0.2)	NA
	N**/Y** YD; N Y**/F** D	3 (15.0)	12 (16.9)	11 (4.3)	11 (12.8)	NA	37 (8.5)	0.0015
	**Y** Y**/F** D; N**/Y F**D; N**/Y** Y**/F** D; N**/Y** Y **D/**Y	2(10.0)	5 (7.0)	8 (3.1)	4 (4.7)	NA	19 (4.4)	0.1928^b^
Sub total		20	71	257	86	NA	434	

^a^A total of 34 isolates with mixed single mutant haplotypes at 184 Y/F were not included. One isolate with a double mutation at 184 and 1246 was included in NFY and the other with mixed mutations was not included

^b^Fisher's exact test was used to evaluate the difference among the groups if 25% of the cells have expected counts less than 5

### Polymorphisms of *pfmdr1*

A total of 434 *P. falciparum* isolates were successfully sequenced and *pfmdr1* allelic variants were observed in only codons 86, 184 and 1246. Twelve haplotypes were identified including six mixed mutant haplotypes. The prevalence of wild type *pfmdr1* NYD was 44.0% (191/434). Comparing the prevalence of *pfmdr1* wild type in the sub-regions of Africa, the region of highest prevalence was southern Africa and the lowest was eastern Africa (*P* < 0.05). Three single mutant haplotypes **Y**YD, N**F**D_,_ NY**Y**, and three double mutant haplotypes of **YF**D, **Y**Y**Y**_,_ and N**FY** were observed. The Y184F of *pfmdr1* was predominant mutation with a prevalence of 24.7% (107/434) and 34 mixed mutant haplotype of N Y/**F** D were confirmed (Table 2). The prevalence of two mixed mutant haplotype N/Y YD and N Y/F D was significant different among the sub-regions (*P* = 0.0015) whereas there were no significant difference of the other *pfmdr1* haplotypes among the groups. The **Y**YD haplotype was at low prevalence of 5.3% (23/434) and not identified in eastern Africa. The single mutant D1246Y variant was not detected and only one isolate carried double mutation N**FY** and the other was mixed mutant haplotype of N/**Y** Y D/**Y**. The **YF**D haplotype (12.7%, 55/434) was more prevalent compared to the other two haplotypes **Y**Y**Y** (0.2, 1/434) and N**FY** (0.2, 1/434). The double mutant haplotype was more common in eastern Africa (25.0%, 5/20) followed by western Africa (18.3%, 13/71) and central Africa (13.2%, 34/257). (Fig. 1).

**Fig. 2:**
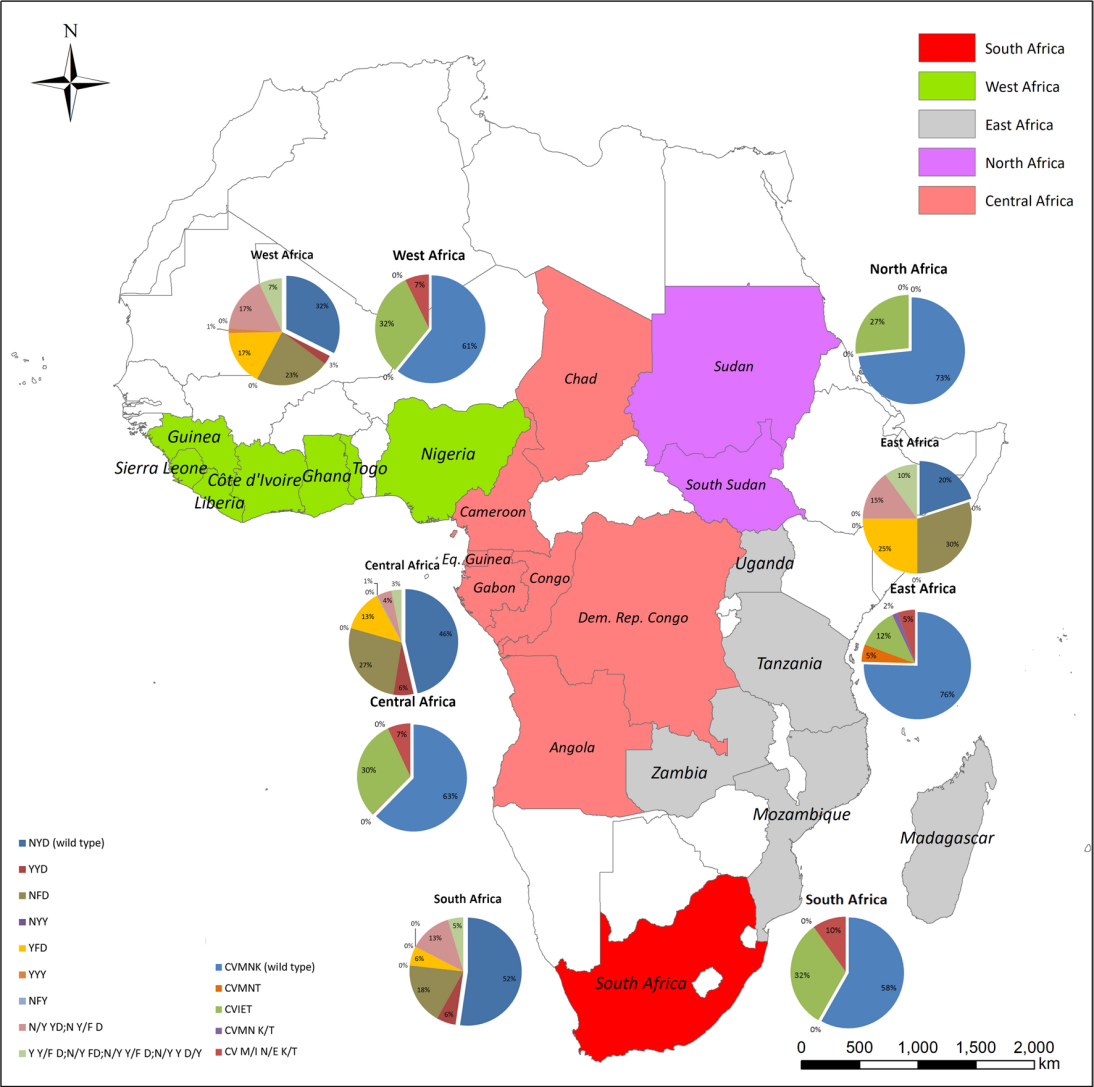
Geographical distribution of *pfcrt* and *pfmrd1* haplotypes in *Plasmodium falciparum* isolates imported from Africa

In addition, six mixed mutant haplotypes (N**/Y** YD, N Y**/F** D, **Y** Y**/F** D, N**/Y F** D, N**/Y** Y**/F** D, and N**/Y** Y D**/Y**) were identified with a combined prevalence of 10.37% (56/434) and the two most common mixed haplotypes were N Y**/F** D and N**/Y** Y**/F** D (Fig. 2 and Table 2).

### Polymorphisms of *pfk13*

**Fig. 3:**
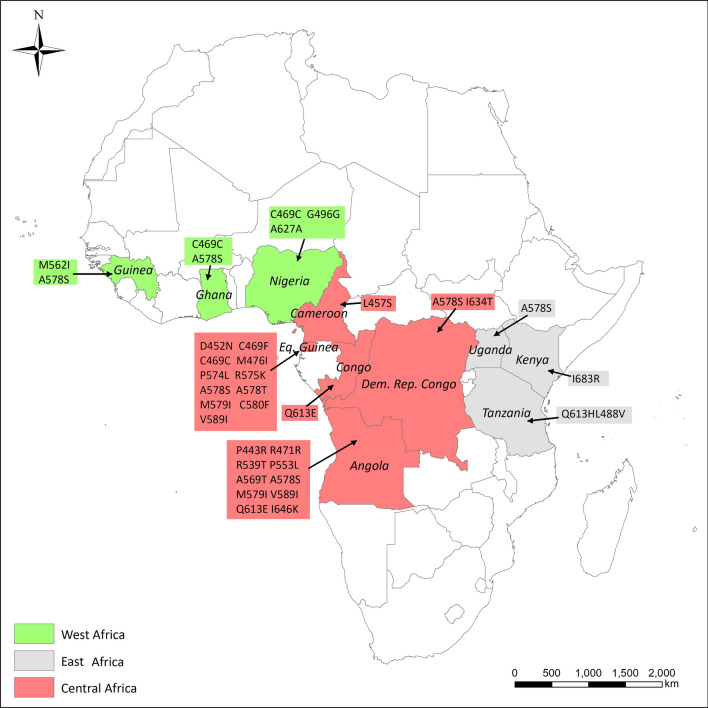
Geographical distribution of *pfk13*-propeller haplotypes in *Plasmodium falciparum* isolates imported from Africa

A total of 1,357 *P. falciparum* isolates from 33 African countries were successfully sequenced of *pfk13*-propeller domain. Twenty-six different mutant alleles were identified including 22 non-synonymous and four synonymous polymorphic sites (Table 3 and Fig. 3). A total of 49 isolates carried single *pfk13* mutation with the prevalence of 3.6% (49/1,357). There were no *pfk13* mutations isolated from northern and southern Africa. The prevalence of *pfk13* mutations was highest in eastern Africa (9.5%, 4/42), followed by central Africa (4.5%, 38/839) and western Africa (1.9%, 7/370). The A578S variant, the most common mutation in *pfk13* in Africa, was identified from 10 isolates (four from Equatorial Guinea, two from Angola, and one each from the Democratic Republic of Congo, Ghana, Guinea, and Uganda.) The Q613E variant was the second-most prevalent mutation, which was found in Angola, Democratic Republic of the Congo and Tanzania. Three mutations associated with artemisinin resistance were identified, including M476I, R539T and P553L. One isolate with R539T (0.1%, 1/1,357) and one with P553L (0.1%, 1/1,357) variant were found from Angola and another isolate with M476I mutation (0.1%, 1/1,357) was from Equatorial Guinea.

**Table 3:** The distribution of *pfk13* mutations in isolates from different countries and geographic regions of Africa

Sub-region of Africa	Source countries	Sample size (N)	No. of samples with *pfk13* mutations	Amino acid of *pfk13* mutations (n)
Central Africa	Equatorial Guinea	224	15	C469C(1) R575K(1) A578S(4) C580F(1) D452N(1) **M476I(1)** V589I(1) P574L(1) A578T(1) M579I(1) C469F(2)
	Congo, DRC	32	1	Q613E (1)
	Republic of Congo	35	2	I634T (1) A578S (1)
	Cameroon	54	1	L457S (1)
	Chad	11	0	
	Central African Republic	3	0	
	Gabon	14	0	
	Angola	466	19	**P553L(1)** A569T(1) A578S(2) Q613E(5) I646K(1) R471R(4) **R539T(1)** P443R(1) V589I(1) M579I(2)
Sub total		839	38	
North Africa	Algeria	1	0	
	Egypt	1	0	
	Libya	3	0	
	Sudan	21	0	
Sub total		26	0	
East Africa	Ethiopia	8	0	
	Kenya	6	1	I683R (1)
	Tanzania	20	2	Q613E (1) L488V(1)
	Uganda	8	1	A578S (1)
Sub total		42	4	
West Africa	Mali	5	0	
	Burkina Faso	2	0	
	Niger	4	0	
	Togo	7	0	
	Ivory Coast	17	0	
	Benin	9	0	
	Liberia	25	0	
	Sierra Leone	29	0	
	Nigeria	152	3	C469C (1) G496G (1) A627A (1)
	Guinea	55	2	M562I (1) A578S (1)
	Ghana	65	2	C469C (1) A578S (1)
Sub total		370	7	
South Africa	Mozambique	28	0	
	Zambia	39	0	
	Malawi	8	0	
	Madagascar	3	0	
	Zimbabwe	1	0	
	South Africa	1	0	
Sub total		80	0	
Total		1357	49	

Three mutations (M476I, R539T, and P553L) associated with artimisinin resistance confirmed by WHO were shown in bold front

## Discussion

This study was part of the national anti-malarial drug surveillance network and supported by the National Malaria Diagnosis Reference Laboratory Network and NMEP. China has set up a well-organized network for malaria diagnosis, treatment and surveillance covering national, provincial and county levels. Nevertheless, there are several challenges in the post-malaria elimination phase in China. One big challenge is how to maintain strong surveillance and response capacity after malaria elimination because thousands of imported malaria cases are reported in China annually. *Plasmodium falciparum* has developed resistance to all anti-malarial drugs, including ACT [22]. This study evaluated the prevalence of *pfcrt*, *pfmdr1* and *pfk13*-propeller mutations of *P. falciparum* isolates imported from Africa and the geographical distribution of the prevalence of these three genes in imported African *P. falciparum* isolates was mapped as well.

CQ was a first-line anti-malarial drug to treat uncomplicated falciparum malaria in Africa from the 1940s, and was widely used because of its high efficacy, safety and low cost [23]. CQ resistance was first identified along the Thai-Cambodian border in the late 1950s [24, 25], and first reported in Africa in the 1970s [26]. In Africa, CQ was replaced by SP and ACT for uncomplicated malaria treatment between the late 1990s and early 2000s. The *pfcrt* mutations in codons 72–76 were considered to be the most reliable molecular marker for CQ resistance [19]. The prevalence of *pfcrt* mutations in Africa decreased significantly in contrast to the late 1990s. The reduction of prevalence of the *pfcrt* mutation and return of CQ sensitivity was also found in other studies in several malaria-endemic countries in Africa [27–29]. The termination of CQ use resulted in recovery of its efficacy. The most common haplotype of *pfcrt* was the wild type CVMNK with the prevalence of 62.8%, which was higher compared with that in the 1990s. Although only a few isolates were detected with single mutation at codon 76, the prevalence of triple mutant haplotype CVIET was 29.4%. In addition, 52 isolates with mixed triple mutant haplotypes CV M**/I** N**/E** K**/T** were identified. According to the published study, CQ resistance may have been caused by selective drug pressure, and multiple genomic background of the strains. Resistant mutations selected by anti-malarial drugs remove linked neutral variation as they sweep (increase in frequency) through a parasite population [30].

The *pfmdr1* gene was associated with resistance to multiple anti-malarial drugs [12–14]. The *pfmdr1* N86Y and *pfcrt* K76T variants have been shown to be in strong linkage disequilibrium, which is associated with CQ, mefloquine, lumefantrine, quinine, and dihydroartimisinin resistance in vitro [31–33]. This study identified *pfmdr1* mutations in only codons 86, 184 and 1246 and total 12 haplotypes, including six mixed mutant haplotypes, were detected. The predominant mutation of Y184F had prevalence of 24.7% (107/434). The single mutant haplotype of *pfmdr1* N86Y was at low prevalence of 5.3% (23/434), lower than another study with the prevalence of 31.0% in 2012 and 8.2% in 2016 [34]. The single mutant type NY**Y** was not detected in this study, suggesting that NY**Y** was rare in Africa compared with previous data [35]. In addition, the single mutant haplotype **Y**YD and N**Y**D was common in Africa while prevalence of the double mutant haplotype **YF**D, **Y**Y**Y** and N**FY** was not significantly different among the different sub-regions (*P* > 0.05). This difference might be caused by the diversity of drug pressure and transmission intensity among the countries or regions in Africa.

Mutations in *pfk13*-propeller domain were first confirmed to be associated with artemisinin resistance in 2014 [20]. Until now, nine validated variants and over 20 candidates or associated mutations of *pfk13* have been identified [6]. Forty-nine out of 1,357 isolates showed *pfk13*-propeller mutations with prevalence of 3.6% (49/1,357) in this study. The non-synonymous mutations in *pfk13* are rare in Africa and their profile is diverse [6, 36–38]. A total of 22 non-synonymous and four synonymous polymorphic sites were identified in this study (Table 3). C580Y and F446I mutations, which are the most common mutations in GMS, and the predominant mutation in southern China, respectively [39], were not detected in imported African isolates in this study. Three mutations in *pfk13*-propeller domain, including M476I, R539T and P553L associated with artemisinin resistance, were observed in three isolates in this study. Another *pfk13* mutation, M579I was identified from one isolate from Equatorial Guinea, which was reported to be associated with artemisinin resistance in Africa [40]. Nevertheless, this mutation was not observed in this study. The presence of C580Y mutation was detected in three patients (2.7%, 3/113) from migrant Chinese workers returning from Ghana in 2013, but this needed further characterization [41]. Previous studies reported that R539T mutation was identified from a population returning to China from Africa [42]. In this study, although one isolate carried the R539T variant, there was no evidence to prove this was an artemisinin-resistant isolate because there was no treatment failure outcome associated with the variant. The A578S variant, which is the most common mutation in *pfk13* in Africa, was identified from 10 isolates (four from Equatorial Guinea, two from Angola, and one each from the Republic of Congo, Ghana, Guinea, and Uganda). A578S is comprised of two tightly linked SNPs and might be involved in artemisinin resistance in Africa [43]. Recently, the de novo emergence and clonal expansion of *pfk13* R561H lineage has been reported in Rwanda and this mutation has been confirmed as a mediator of artemisinin resistance in vitro [44]. Another more recent study reported that *pfk13* R561H occurred in 4.5% (3/66) of the isolates collected in southern Rwanda in 2019 [45]. Interestingly, an imported malaria case from Rwanda to China was detected with R561H mutation [46] and one isolate from southeast Tanzania carried this mutation too [47]. Therefore, molecular marker surveillance could provide early warning and evidence for efficacy of anti-malarial drugs to treat imported cases. China has set up an anti-malarial drug surveillance network that is responsible for implementing an integrated drug efficacy study (iDES) of anti-malarial drugs for national policy and molecular surveillance in the entire country.

## Limitations

This study only evaluated the prevalence of molecular markers associated with anti-malarial drug resistance of imported cases from Africa and the treatment outcome was not analysed. All imported malaria cases will be treated according to national anti-malarial drug policy (Additional file 3). The iDES, as one component of routine surveillance systems, will be considered in the malaria elimination phase to provide evidence for updating the guidelines of anti-malarial drug treatment in China, especially for imported malaria cases. In addition, although mixed haplotypes were identified in some samples, the multiplicity of infection of the samples was not tested in this study.

## Conclusion

This study provides evidence to give insight into potential issues with anti-malarial drug resistance to inform national anti-malarial drug policy in China to treat imported cases.

## Supplementary Information

**Additional file 1: Table S1.** Primer sequences and nested PCR amplification conditions for *pfcrt*, *pfmdr1* and *pfk13 *genes in* Plasmodium falciparum*.

**Additional file 2: Fig. S1.** Indigenous malaria case distribution at county level in China, 2012–2015.

**Additional file 3. **Anti-malarial drug policy of China.

## Data Availability

The datasets analysed in this study are available from the corresponding author on reasonable request.
