# Supplementary material for: Molecular surveillance of pfcrt, pfmdr1 and pfk13-propeller mutations in Plasmodium falciparum isolates imported from Africa to China
Source: Malar J. 2021 Feb 6;20:73. doi: 10.1186/s12936-021-03613-5 (PMC7866736; doi:10.1186/s12936-021-03613-5)
Supplement: Supplementary file 1 — Additional file 1: Table S1. Primer sequences and nested PCR amplification conditions for pfcrt, pfmdr1 and pfk13 genes in Plasmodium falciparum. [file 12936_2021_3613_MOESM1_ESM.docx]

**Table S1 Primer sequences and nested PCR amplification conditions for *pfcrt*, *pfmdr1* and *pfk13* genes in *Plasmodium falciparum***

| **Genes** | **Primer sequences (5'-3')** | **Amplification conditions** | **Products size (bp)** | **References** |
| --- | --- | --- | --- | --- |
| *Pfcrt* | CRT-1F: ccgttaataataaatacacgcag  CRT-1R: cggatgttacaaaactatagttacc | 95°C × 5 min; 30 cycles of 92°C × 30 sec, 56°C × 30 sec, 60°C × 1 min; 60°C × 3 min | 537 | [19] |
|  | CRT-2F: tgtgctcatgtgtttaaactt  CRT-2R: caaaactatagttaccaattttg | 95°C × 5 min; 30 cycles of 92°C × 30 sec, 48°C × 30 sec, 65°C × 30 sec; 60°C × 3 min | 145 |  |
| *Pfmdr1* | MDR1-1F: ttaaatgtttacctgcacaacatagaaaatt  MDR1-1R: ctccacaataacttgcaacagttctta | 95°C × 3 min; 35 cycles of 93°C × 30 s, 52°C × 30 s, 72°C × 1 min; 72°C × 5 min | 612 | [21] |
|  | MDR1-2F: tgtatgtgctgtattatcagga  MDR1-2R: ctcttctataatggacatggta | 95°C×3 min; 35 cycles of 93°C × 30 s, 52°C × 30 s, 72°C × 1 min; 72°C × 5 min | 526 |  |
|  | MDR2-1F: aatttgatagaaaaagctattgattataa  MDR2-1R: tatttggtaatgattcgataaattcatc | 95°C × 3 min; 35 cycles of 93°C × 30 sec, 52°C × 30 sec, 72°C × 1 min; 72°C × 5 min | 880 |  |
|  | MDR2-2F: gaattattgtaaatgcagcttta  MDR2-2R: gcagcaaacttactaacacg | 95°C × 3 min; 35 cycles of 93°C × 30 sec, 52°C × 30 sec, 72°C × 1 min; 72°C × 5 min | 799 |  |
| *Pfk13* | K1-F: cggagtgaccaaatctggga  K4-R: gggaatctggtggtaacagc | 95^o^C × 2 min; 30 cycles of 95^o^C × 30 sec, 60^o^C × 90 sec, 72^o^C × 90 sec; 72^o^C × 10 min | 2097 | [20, 38] |
|  | K2-F: gccaagctgccattcatttg  K3-R: gccttgttgaaagaagcaga | 95^o^C×2 min; 30 cycles of 95^o^C × 30 sec, 60^o^C × 90 sec, 72^o^C × 90 sec; 72^o^C × 10 min | 850 |  |
