# Supplementary figures and images for: Molecular surveillance of pfcrt, pfmdr1 and pfk13-propeller mutations in Plasmodium falciparum isolates imported from Africa to China
Source: Malar J. 2021 Feb 6;20:73. doi: 10.1186/s12936-021-03613-5 (PMC7866736; doi:10.1186/s12936-021-03613-5)

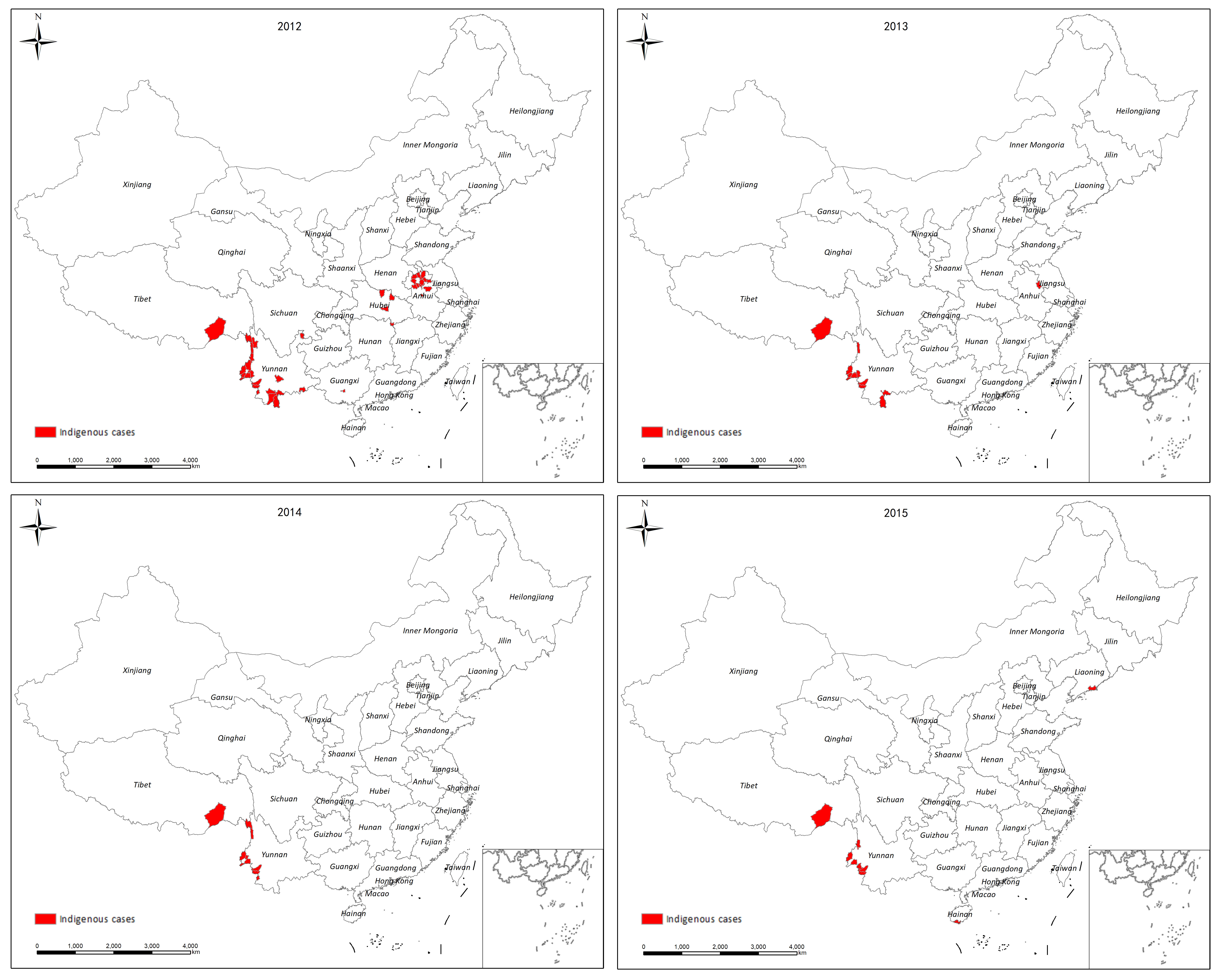

Supplement: Supplementary file 2 — Additional file 2: Fig. S1. Indigenous malaria case distribution at county level in China, 2012–2015. [file 12936_2021_3613_MOESM2_ESM.jpg]
