# Supplementary material for: Molecular surveillance of pfcrt, pfmdr1 and pfk13-propeller mutations in Plasmodium falciparum isolates imported from Africa to China
Source: Malar J. 2021 Feb 6;20:73. doi: 10.1186/s12936-021-03613-5 (PMC7866736; doi:10.1186/s12936-021-03613-5)
Supplement: Supplementary file 3 — Additional file 3. Anti-malarial drug policy of China. [file 12936_2021_3613_MOESM3_ESM.docx]

**Anti-malarial drug policy in China (WS/T 485-2016)**

All laboratory confirmed malaria cases should be treated with standard anti-malarial treatment. For specific treatment plan, please refer to the industry standard "Specification for The Use of Anti-malarial Drugs (WS/T 485-2016)".

**1.Treatment of *P. vivax* and *P. ovale* cases**

The first choice is chloroquine phosphate plus primaquine phosphate for 8 days, or an artemisinin combination therapy (ACT) based on artemisinin drugs plus primaquine phosphate Tablets for 8 days. The commonly used ACT drugs include dihydroartemisinin piperaquine Tablets, artesunate amodiaquine Tablets plus artemisinin piperaquine Tablets. Before the next epidemic season, the patients were treated with primaquine phosphate for 8 days.

**2. Treatment of *P. malariae* cases**

The patients were treated with chloroquine phosphate Tablets for 3 days or oral ACT.

**3. Treatment of falciparum malaria cases**

The patients were treated with oral ACT.

**4. Treatment of severe malaria cases**

Artesunate injection is the first choice for intravenous injection, and artemether intramuscular injection or pyronaridine phosphate intravenous drip can be used. At the same time, symptomatic treatment and supportive therapy were given to reduce complications and complicated infection. The patients were treated with artemisinin injection for at least 7 days. According to the "Professional Consensus on Anti-malarial Treatment and Malaria Prevention" issued by the general office of the National Health Commission No.7, artemisinin injections can be used for malaria treatment in hospitalized patients. After the patients' condition is relieved and they can eat on their own, they can use ACT orally for another course of treatment.
